# Expected Future Subjective Social Status Moderates the Relations between Perceived Parental Expectation and Persistence among Chinese Rural Adolescents

**DOI:** 10.3390/bs14080722

**Published:** 2024-08-17

**Authors:** Feng Zhang, Rui Yang, Xiaodan Xu

**Affiliations:** 1 Psychological Research and Counseling Center, Southwest Jiaotong University, Chengdu 610031, China; zhangfpsy@swjtu.edu.cn (F.Z.); florayang1987@163.com (R.Y.); 2 School of Arts and Communication, Beijing Normal University, Beijing 100875, China; 3 Documentary Center, Beijing Normal University, Beijing 100875, China

**Keywords:** parental expectations, future subjective social status, persistence, rural adolescents

## Abstract

Adolescents’ expectations on future subjective social status (SSS) may play a critical role in the relations between perceived parental expectations and persistence; however, there is a lack of research exploring this effect in the context of families experiencing greater economic risk. This study aimed to explicitly address this issue. A total of 698 Chinese rural adolescents participated in this study (*M*_age_ = 13.32 years; 54.60% boys). The results showed that for rural adolescents with lower expected future SSS, perceived parental expectation was negatively related to persistence; for rural adolescents with higher expected future SSS, perceived parental expectation was not significantly correlated with persistence. These findings imply the adverse effects of high perceived parental expectation on rural adolescents’ persistence and that expected future SSS can alleviate this adverse relationship.

## 1. Introduction

Expectations are predictions and beliefs that individuals have regarding future outcomes. Adolescents’ perceived expectations of significant others (e.g., parents) and their own expectations are both theoretically assumed to affect their behaviors and academic performance [1]. Previous research has revealed the effects of perceived parental expectation and adolescents’ own expectations separately. For example, when adolescents perceive their parents to have higher expectations for their education and careers, they take more action and exert more effort to meet these expectations [2]. Positive results were obtained as for adolescents’ own expectations. When adolescents expect to have a future with higher socioeconomic status (e.g., higher education and well-paid job), they exhibit more perseverance in the face of challenges [3,4]. However, less research has explored these two sources of expectations simultaneously. Moreover, as for perceived parental expectation, in addition to its positive effects, its negative effects were also found [5], which may be detrimental to persistence. This inconsistent result suggests that a moderator may play in the link between perceived parental expectation and persistence. Therefore, this study explored these two sources of expectations to reveal their individual effect on persistence, and to uncover whether adolescents expected future subjective social status (SSS) could be the moderator.

In particular, adolescents in Chinese rural families were targeted to explore the above associations. This is partly because Chinese parents frequently express high expectations for their children to facilitate positive developmental outcomes [6]. Moreover, China’s household registration system generally divides residents into rural and urban Hukou groups [7,8], and families with rural Hukou are usually more economically disadvantaged than families with urban Hukou. This may result in rural adolescents falling behind their urban peers in terms of behavioral and academic outcomes [9,10]. According to the data from the seventh national census in 2020, more than one-third of children and adolescents live in rural areas (accounting for approximately 37% of national peers). A positive developmental outcome for such a large group is vital to the well-being of society as a whole. Rural adolescents with greater family economic risk may face more difficulties and challenges; thus, they need to expend more effort and persevere to achieve their goals. Greater perseverance can increase the likelihood of escaping impoverished environments, which has obvious practical value for rural adolescents [11]. As such, it is necessary to investigate the factors associated with rural adolescents’ persistence, which is an important psychological assistance path to promote their positive outcomes.

Furthermore, the parental expectation that this study focuses on is a concept based on traditional Chinese culture, and its effect may differ between rural and urban contexts. Despite the fact that both collectivistic and individualistic values coexist in China, the rural context maintains more collectivistic values, emphasizing interdependence, obligation, and obedience in the family [12]. In this way, rural adolescents may be more likely than urban adolescents to obey their parents’ authority and meet their parents’ expectations. Thus, rural adolescents’ perceived parental expectations may help them obtain better developmental outcomes, such as maintaining greater persistence in the face of difficulties. However, during the process of urbanization, individualistic values are gradually becoming more popular [13,14]. Rural adolescents may also be exposed to more information about individualism in the process of socialization, which may lead them to pay attention to their own choices and autonomy and care about whether their expectations can be realized. As such, rural adolescents’ own expectations may be a more important factor motivating them to take actions (e.g., persistence and effort) to achieve their personal goals. Nevertheless, less is known about the associations among adolescents’ perceived parental expectation, their own expected future SSS, and their persistence in rural contexts from the perspective of cultural value change. Therefore, this study aimed to address this issue, which can also provide evidence for the impact of changes in social values on individual development [15].

### 1.1. The Relations between Perceived Parental Expectation and Adolescent Persistence

Persistence is regarded as a motivational resource [16] and involves individuals’ enduring participation and effort in the face of challenged and difficult tasks [17]. Adolescents with greater persistence may work hard and concentrate on the tasks at hand, and they will never give up even if they encounter challenging tasks [18]. Unfortunately, rural adolescents may face greater economic risks and exhibit weaker persistence [19,20]. Uncovering the factors that contribute to persistence would be of significance for rural adolescents, as it could provide evidence of how to cultivate rural adolescents’ persistence to help them overcome challenges and difficulties, which in turn increases their chance for a better life.

Parental expectations are parents’ beliefs regarding their children’s future achievement in several important developmental fields, such as education and occupation [21,22]. Parents have high expectations for their children worldwide, especially in East Asian countries [23]. Shaped by traditional Confucian culture, Chinese families have formed a family culture centered on Confucianism, emphasizing core values such as human malleability, self-improvement, and family obligation [24]. As such, in the context of Chinese culture, parents are expected to have greater expectations for their children’s future, to motivate their children to strive for a bright future and to fulfill family obligations [11]. The famous Chinese saying “expected my child to be a dragon” directly reflects the great value of Chinese parents’ expectations for their children.

The ubiquity of parental expectations might inevitably correlate with adolescents’ developmental outcomes. According to the Chinese family socialization model [11,25], parents who have greater expectations for their children’s future would demonstrate more behavioral towards their children’s development, thus promoting the outcomes of their children. However, a previous study has suggested that it is the supportive process of parents that facilitates adolescents’ development, rather than just expressing high expectations for them [26]. In addition, relevant research has shown mixed results regarding the relations between parental expectations and adolescent developmental outcomes. This leads to the question of whether perceived parental expectations effectively promote adolescent persistence in the face of difficulties and setbacks.

Some evidence has shown the positive effects of parental expectations [27] and there was evidence that parental expectations were positively associated with academic performance [28,29]. When adolescents perceive higher parental expectations, they are more likely to engage in plans that meet their parents’ expectations [2], which also implied the positive effects of adolescents’ perceived parental expectations on behavioral outcomes. But other research has suggested that perceived parental expectations might not always be beneficial to adolescents’ behavioral outcomes. Adolescents who report higher parental expectations may experience greater test stress [5], which in turn might reduce their persistence in tasks [30]. In addition, parental expectations are not positively related to parental support, while effective help from parents is a potent factor in promoting perseverance in the face of difficulties [31]. Moreover, recent evidence revealed a nonsignificant correlation between parental expectations and academic outcomes for individuals who experienced greater economic risk [32]. Considering that persistence is always positively related to academic performance [33], it is possible that parental expectations are not significantly associated with adolescents’ persistence.

Taken together, although related evidence has implied that there is a mix of results of perceived parental expectation and persistence, there is less research directly regarding this relationship in rural adolescents. Thus, this study aimed to clearly reveal the correlation between perceived parental expectations and rural adolescents’ persistence. Meanwhile, considering the potential mixed results above, there might be moderators in the link between perceived parental expectation and persistence; this study has explicitly addressed this issue.

### 1.2. Potential Moderating Role of Expected Future Subjective Social Status

Subjective social status (SSS) is an individual’s perceived socioeconomic position on a social ladder; it comprises education, occupation, and income level [34,35]. Accordingly, expected future SSS is defined as an individual’s anticipation of socioeconomic standing on a social hierarchy in the future; thus, it determines the social class that adolescents expect to belong to as well as their future-oriented thoughts. The positive effects of expected SSS on adolescents’ developmental outcomes have been well documented [36,37,38]. Furthermore, according to the prospect theory, future expectations can motivate adolescents to take actions and thus facilitate behavioral outcomes, such as persistence or effort [39,40,41,42]. Moreover, adolescents who were cued with positive expectations about the future in the experimental situation were found to be more perseverant in challenging tasks [43]. Taken this evidence together, adolescents’ expected SSS in the future might by positively correlated with their behavioral outcomes (e.g., persistence).

In addition to its direct effects on persistence, expected future SSS might also act as a possible moderator in the link between perceived parental expectations and adolescents’ persistence. In fact, adolescents do not receive their parents’ beliefs and values (e.g., expectations) passively [44], and adolescents’ own expectations can be different from their perceived parental expectations [45]. This may be especially true in adolescence, a period that eagerly demands autonomy [46]. Adolescents may prioritize their own expectations over those of their parents. According to expectancy–value theory, individuals’ expectations for success and the extent to which they value a given task are closely related to persistence [1]. In accordance with this theory, adolescents are likely to possess greater motivation when themselves expect to do well and when the tasks have some value to them [3]. In addition, when adolescents in low-income families have higher expectations for the future, they exhibit more effortful behaviors [47]. As such, compared with the fulfillment of significant others (e.g., parents), adolescents’ own expectations might play a vital role in the prediction of their persistence.

Although there is no direct evidence to demonstrate the moderation effect of adolescent expected SSS, relevant evidence can provide support for this. First, related evidence has shown that adolescents are motivated when they want to achieve academic success, irrespective of their parents’ beliefs [48]. As such, it is possible that adolescents with higher expected SSS had stronger persistence, and parental expectations have less impact on their persistence. Second, similar related evidence has shown that when adolescents perceive their parental expectations to be higher than their own, they have worse academic outcomes [49]. Thus, negative effects that are related to worse academic performance (e.g., weaker persistence) may also occur for adolescents who perceive their parents’ expectations to be higher than their own. However, less is known about how adolescent expectations of future SSS interplay with their perceptions of parental expectations in relation to the persistence in rural adolescents.

### 1.3. The Current Study

Expectations have long been used to explain adolescents’ behavioral outcomes. Both perceived parental expectation and adolescents’ own expectation for socioeconomic status in the future might be important sources of motivation for them to persist. In Chinese culture, higher perceived parental expectations are assumed to motivate adolescents to exert more effort and thus to be more likely to succeed in life, which is especially true for rural families. However, relevant empirical evidence regarding the relationship between perceived parental expectation and persistence is inconsistent.

**Figure 1:**
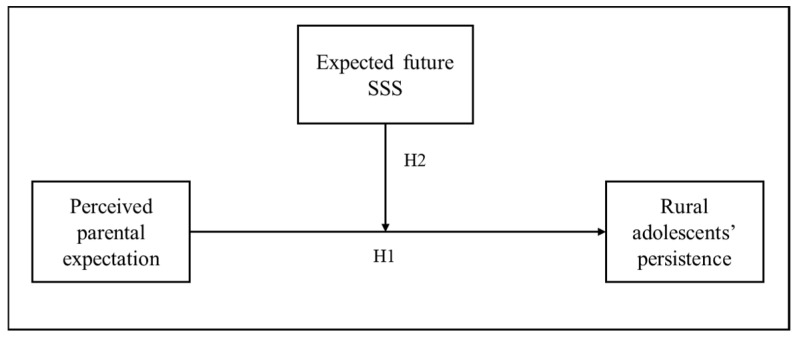
Conceptual model of expected future SSS as a moderator of the link between perceived parental expectation and persistence in rural adolescents.

This study contended that adolescents’ own expectations of future SSS might play a role in the relationship between perceived parental expectation and their persistence. Therefore, this study focused on adolescents in rural Chinese families to explicitly reveal the relations between perceived parental expectation and adolescents’ persistence and to explore whether adolescent expected future SSS moderates this link. Figure 1 graphs the hypothesized model of the present study to clarify the relationships among the main variables. Based on this model, two research questions were aimed to be examined by using cross-sectional data:

(1) Research question 1: Are perceived parental expectations significantly correlated with persistence among Chinese rural adolescents?
(2) Research question 2: Whether expected future SSS significantly moderates the link between perceived parental expectation and rural adolescents’ persistence?

In addition, adolescents’ gender and age were considered covariate variables in this study. Previous studies have suggested that engagement decreases with increasing age in adolescence [50,51]. Moreover, gender differences in adolescents’ engagement have also been shown [52]. Given that adolescent persistence, which this study focused on, is a concept related to behavioral engagement, it is possible that gender and age may also be relevant to persistence. Thus, gender and age were regarded as covariate variables and controlled for in subsequent data analyses.

## 2. Materials and Methods

### 2.1. Participants

A middle school was selected from a town that was located in a county in Chongqing Municipality in China by using convenient sampling. All the students were from local villages and towns, and therefore, their families had rural or urban household registration (Hukou). Meanwhile, the county was once a national poverty-stricken area, with a relatively low level of economic development. The target sample with rural Hukou in this school was invited to participate in this study. Prior to data collection, a power analysis was conducted (*f*^2^ = 0.02, *α* = 0.05) using G*Power 3.1, and it recommended that a minimum sample size of 550 was needed to detect a power of (1 − *β* = 0.80) in multiple regression with three predictors [53]. A total of 698 rural adolescents participated in this study (*M*_age_ = 13.32 years, *SD* = 0.72, 54.60% boys), including 403 seventh-grade and 295 eighth-grade adolescents. The number of samples collected in this study met the above recommended sample size standards to achieve a power of 0.80.

In addition, most adolescents (*n* = 633, 90.69%) were from two-parent families, and a small proportion of adolescents (*n* = 65, 9.31%) were from single-parent families (i.e., divorce of parents or one of the parents passed away). Moreover, most adolescents reported that they were from non-one-child families (86.30%), and the remaining adolescents (13.70%) were from one-child families.

### 2.2. Procedure

This was a quantitative cross-sectional study, and the data were obtained directly from the school administration and through adolescents’ reports. The investigation protocol was approved by the local educational department and the administration of the school prior to data collection. In addition, prior to participation, all participants were informed that their participation was voluntary and that they could withdraw at any time. First, information on the students’ family Hukou was obtained from the records of the school administration. Then, a list of rural students was compiled by the researchers based on the above information. Second, rural adolescents in seventh- and eighth grades were invited to participate in this study. To protect the privacy of the participants and to ensure the simplicity of the data arrangement, each invited participant was named with a numerical code in advance. The adolescents completed anonymous questionnaires with their own corresponding numbers in quiet classrooms, and the questionnaires included demographic information, perceived parental expectations, persistence, and expected future SSS. After the completion of the survey, each adolescent received a gift (approximately $1.5) for his or her participation.

### 2.3. Measures

#### 2.3.1. Perceived Parental Expectation

A subscale of the Living-Up-to Parental Expectation Inventory (LPEI) [22] was used to assess adolescents’ perceived parental expectations for education and career development. This subscale consists of nine items (e.g., “parents expect me to have excellent academic performance”, “parents expect me to pursue their ideal careers”), and adolescents reported how strongly they agreed with each item on a scale ranging from 1 (not at all expected) to 6 (very strongly expected). All items were positively scored, and higher scores represent higher levels of perceived parental expectations in the field of education and career. The reliability of this scale was adequate, and the Cronbach’s alpha was 0.86 in the present study.

#### 2.3.2. Future Subjective Social Status (SSS)

A 10-runged ladder representing socioeconomic status is frequently used to measure adolescents’ current SSS [54]. The use of single-item measures to access concrete constructs is commonly accepted, and there is no predictive validity between single-item and multiple-item measures [55,56], and single-item measures can reduce the burden on respondents [57]. Moreover, previous studies have adapted this tool to assess adolescents’ SSS when they grow up [58,59]. Accordingly, adolescents in this study were asked to report where they expected themselves to stand on the 10-runged ladder within 10 years (*M*_ladder_ = 7.34, *SD* = 1.92). Higher scores represent higher levels of expected SSS in the future.

#### 2.3.3. Persistence

Adolescents’ persistence was measured by five items adopted from the OECD Programme for International Student Assessment (PISA) surveys [60]. The PISA surveys collect data on 15-year-old adolescents in participating countries (e.g., China) every three years. The items about persistence were first added in 2012, and they demonstrated acceptable reliability for Chinese students. These items were also included and used to measure persistence in the latest PISA survey [61]. In this study, minor modifications were made to the original two reverse-scored items to ensure that each item was a positive statement. The adolescents responded on a scale ranging from 1 (not at all like me) to 5 (very much like me) (e.g., “when confronted with a problem, I give up easily”; “I continue working on tasks until everything is perfect”). The mean scores of all items were calculated, with higher scores representing higher levels of persistence. The Cronbach’s alpha was 0.89 in the present study.

### 2.4. Data Analyses

All the data analyses were conducted using SPSS 25.0 and Mplus 7.4. First, the descriptive statistics and correlations of the main variables were analyzed using SPSS 25.0. Specifically, Spearman correlation analysis was used to calculate the correlation coefficient between the study variables. Second, Mplus 7.4 [62] was used to test the moderating effects of future SSS on the relationship between perceived parental expectations and persistence, with the full information maximum likelihood (*FIML*) method addressing the missing data.

Specifically, structural equation modeling (SEM) was used to test the moderation model. The independent variables (i.e., perceived parental expectation and expected future SSS), the interaction term (i.e., perceived parental expectation × expected future SSS), and covariate variables (i.e., gender and age) were added to the model. Then, a moderation model with rural adolescents’ persistence as the dependent variable was run. Moreover, the MODEL CONSTRAINT command was used to compute the effects of perceived parental expectation on persistence at higher and lower levels of expected future SSS. The following model fit indices were employed to evaluate an adequate model fit: the comparative fit index (*CFI*), Tucker–Lewis index (*TLI*), and root mean square error of approximation (*RMSEA*) indices. In addition, 5000 bootstrapping resamples with 95% confidence intervals were generated, and the confidence intervals are presented. A moderating effect is considered to be effective when the *p*-value is significant (*p* < 0.05) and the confidence interval does not include a zero.

Moreover, adolescents’ gender and age were controlled for in the analyses. Gender was transferred into a dummy variable (boy = 0, girl = 1), and age was z-transformed in the SEM analysis. All the other variables were standardized to z scores to reduce collinearity, and the interaction term was calculated from these z scores. Finally, the slope coefficients at 1 standard deviation (*SD*) above and 1 standard deviation (*SD*) below the mean of the expected future SSS were graphed when the moderating effect was significant.

## 3. Results

### 3.1. Descriptive Statistics and Correlations between the Main Variables

Table 1 presents descriptive statistics and provides details on the score, range, mean, and standardized deviation of each study variable. Initially, whether these variables satisfied the conditions for using the Pearson correlation analysis was examined. In particular, gender was a dummy variable and therefore was regarded as a continuous variable in the data analyses, and all other variables were continuous. A linear relationship between each pair of these variables (i.e., perceived parental expectation, persistence, and expected future SSS) was found via scatter plots. Moreover, the Shapiro–Wilk test was conducted to test the normality of continuous variables, and the results revealed that the variables did not follow a bivariate normal distribution (*ps* < 0.05). As such, the data did not have a normal distribution, and Pearson correlation analysis was not applicable. Thus, this study used Spearman correlation analysis to test for correlations between variables.

Spearman correlation analysis was used to calculate the correlation coefficient between the study variables, and Table 2 presents the correlations. The results showed that perceived parental expectations were not significantly related to either future SSS (*r* = 0.05; *p* = 0.154) or adolescent persistence (*r* = 0.01; *p* = 0.930). In addition, future SSS was significantly correlated with adolescent persistence (*r* = 0.25; *p* < 0.001).

### 3.2. The Moderating Effects of Expected Future SSS

**Figure 2:**
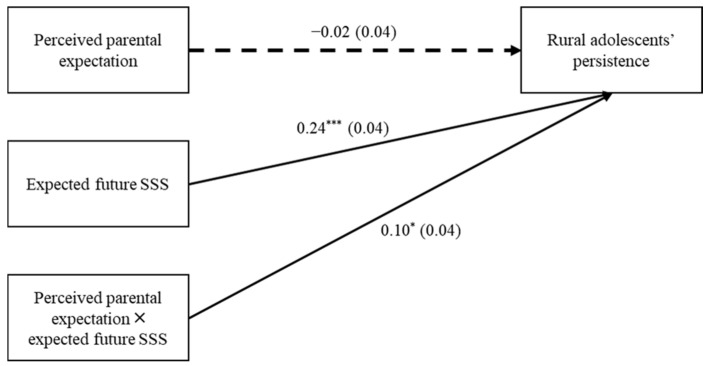
Main results of the moderation model. The solid lines indicate significance at *p*-values less than 0.05 (*p* < 0.001 and *p* = 0.012, respectively), and the dotted line indicates non-significance at *p*-value greater than 0.05 (*p* = 0.556). The standardized coefficients are outside of the brackets, and the standard errors are inside the brackets. Gender and age were controlled for but are not shown in the figure for simplicity; SSS = subjective social status. *** *p* < 0.001, and * *p* < 0.05.

The moderation model with persistence as the dependent variable was tested. This model assessed the main effect of perceived parental expectation on persistence and whether expected future SSS moderated this effect. Table 3 and Figure 2 present the moderating results of adolescents’ expected future SSS after controlling for gender and age.

The model fit indices were acceptable: *χ^2^*_(6)_ = 14.50, *CFI* = 0.89, *TLI* = 0.90, *RMSEA* = 0.04 [0.02, 0.07]. First, the results showed that perceived parental expectation did not significantly predict adolescents’ persistence (*β* = −0.02, *p* = 0.556, *95% CI* = [−0.10, 0.06]), indicating that there may be no correlation between perceived parental expectation and persistence. Second, expected future SSS significantly and positively predicted adolescents’ persistence (*β* = 0.24, *p* < 0.001, *95% CI* = [0.16, 0.32]), indicating that expected future SSS has a positive effect on persistence. In addition, the interaction term of perceived parental expectation and expected future SSS significantly and positively predicted adolescents’ persistence (*β* = 0.10, *p* = 0.012, *95% CI* = [0.02, 0.17]), indicating that expected future SSS moderated the relationship between perceived parental expectation and persistence.

**Figure 3:**
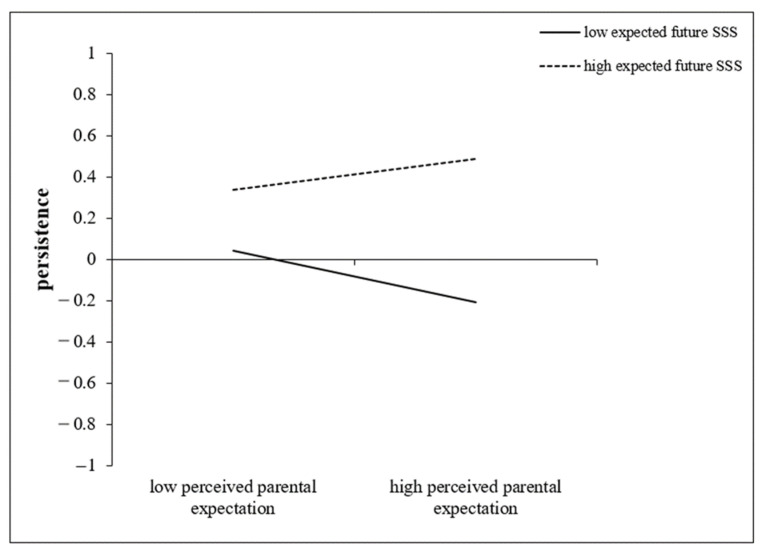
The moderating role of expected future SSS. Two levels of future SSS are graphed: one standard deviation (*SD*) above the mean and one standard deviation (*SD*) below the mean; the solid lines indicate significance at *p*-value less than 0.05 (*p* = 0.005), and the dotted line indicates non-significance with *p*-value greater than 0.05 (*p* = 0.103); SSS = subjective social status.

To better present the significant moderating effect of expected future SSS in the moderation model, the simple slopes of perceived parental expectation on rural adolescents’ persistence at lower (−1 *SD*) and higher (+1 *SD*) levels of expected future SSS were graphed (Figure 3).

The results indicated that perceived parental expectation was not significantly related to the persistence of adolescents with higher future SSS (*simple slope* = 0.07, *t* = 1.63, *p* = 0.103, *95% CI* = [−0.04, 0.19]). Moreover, perceived parental expectation was negatively correlated with the persistence of adolescents with lower future SSS (*simple slope* = −0.13, *t* = −2.80, *p* = 0.005, *95% CI* = [−0.22, −0.03]). As such, rural adolescents who have lower expected future SSS are more likely to experience the negative effects of perceived parental expectation on their persistence.

## 4. Discussion

Expectations are future-oriented thoughts that fuel adolescents’ motivations in the face of challenges and difficulties, implying that there may be positive relationships between expectations and behavioral outcomes. Chinese rural adolescents are expected by their parents to bring honor to their families and improve their families’ economic conditions. Moreover, adolescents are expected to achieve a prosperous future not only by parents but also by themselves, yet how the two sources of expectations interplay to guide adolescents’ actions (e.g., persistence) remains unknown. Therefore, this study explicitly addressed the associations among perceived parental expectation, adolescents’ own expectations of future SSS, and persistence in the context of Chinese rural families. As a result, this study revealed a positive effect of adolescents’ own expectations of future SSS on persistence but did not find a significant correlation between perceived parental expectation and persistence. In addition, expected future SSS played a moderating role in the relationship between perceived parental expectation and persistence. When rural adolescents expected higher future SSS, perceived parental expectation was not related to persistence. When rural adolescents expected lower future SSS, the greater the perceived parental expectation, the weaker the persistence.

Consistent with expectancy–value theory [1], this study found that adolescents’ expectations for future SSS were positively correlated with persistence. Meanwhile, this finding is consistent with both the SSS theory and the prospect theory, which suggest that future SES positions affect adolescents’ behavioral outcomes [39,63,64,65]. For rural adolescents with higher expected future SSS, they demonstrated stronger persistence; in other words, perceived parental expectation played less effect on their persistence. This finding suggested the importance of adolescents’ fulfillment of their own rather than the significant others’ expectations in predicting persistence, reflecting a self-fulfilling prophecy. For rural adolescents with higher expected future SSS, they have higher persistence that might be associated with better academic performance [17], which is a major predictor of higher socioeconomic position, especially for rural families. As such, these adolescents’ expectations of higher SSS may be realized in the future. Similarly, adolescents who expect lower levels of SSS reduce their effort and perseverance, which might be related to poor learning outcomes. In this way, those adolescents might have difficulty attaining higher future socioeconomic positions in the rural context, and ultimately, their low expectations are more likely to be confirmed in the future.

In addition, an expectation for future SSS might meet adolescents’ autonomy needs [66,67], which may increase adolescents’ perseverance. This might be especially true for adolescents, as adolescence is a period that demands autonomy and asserting independence from one’s parents [46]. In this way, when adolescents themselves hold the view that higher future goals (i.e., higher SES positions in the future) await them, they demonstrate greater persistence to fulfill those goals. Moreover, research has suggested that a lack of control causes adolescents from poor families to demonstrate weaker perseverance [20,68], while expectations for future SSS are positively related to self-control abilities [65]. As such, for rural adolescents, possessing higher future expectations on SSS may provide potent motivational resources that exactly remedy the lack of self-control that poor environments elicit for them. Thus, rural adolescents with higher expected future SSS manifested stronger persistence irrespective of the degree to which their perceived parental expectations.

However, this finding did not provide supports for the Chinese family socialization model [11] in the context of rural families and did not find a positive correlation between perceived parental expectation and adolescent persistence. Moreover, when adolescents themselves had lower expected SSS, perceived parental expectation even had an adverse effect on their persistence. As such, although some research has revealed the positive effect of perceived parental expectations [2], similar results were not found in this study. This finding is in line with the suggestions of previous research [6,21], indicating the potential adverse effects of perceived parental expectation for rural adolescents. In this way, considering the ubiquity of Chinese parents’ high expectations for their children, this finding has added to a growing awareness that high parental expectations can have a negative effect in the context of rural families.

As this study indicated, parental expectations that emphasize the achievement of family honor and the obedience of parental authority, such a traditional cultural value, have no significant positive effect on rural adolescents’ persistence. A previous study suggested that rural adolescents are more likely to obey parental authority than are urban adolescents [69]; thus, rural adolescents may be more likely to meet their parents’ expectations through effort and persistence. However, this study indicated that even rural adolescents were not more persistent in the face of difficulties and setbacks because of their parents’ high expectations. With the change in cultural value in China [13,14], the younger generation may be more of an advocate of personal culture, emphasizing personal concerns, independence, and autonomy. As such, rural adolescents may be more concerned about fulfilling their own expectations than about meeting family expectations and demands. Moreover, when perceived parental expectations are at odds with their own, adolescents may be more likely to prioritize their own expectations. Thus, adolescents’ own expectations play a more important role in facilitating persistence. These findings suggested that it is necessary for parenting practices to keep up with changes in social values. If parents blindly express high expectations and demands for the younger generation to bring honor to the family, hoping to inspire their motivation, this may have little effect and even have negative effects.

In the context of rural families, especially for the participants in this study, which targeted adolescents who lived in a once poverty-stricken area whose parents had limited resources (e.g., poor education capital, chaotic living environments, and inappropriate parenting) that are difficult to support their children’s development [70,71]. More importantly, rural parents might also be confused about how to effectively facilitate their children’s positive outcomes and have less involvement for their children [72]. Parents may frequently hold high expectations for their children; however, they do not provide effective assistance to their children. Therefore, rural adolescents who perceive high parental expectations but receive less effective support may fail to persist towards their goals, particularly for those who have lower levels of future expectations on SSS.

Moreover, higher perceived parental expectations are associated with higher levels of stress, which might reduce adolescents’ persistence. As indicated by previous evidence, adolescents who perceived higher parental expectations reported more stressful experiences [5], which were exactly correlated with lower levels of persistence [30,73,74]. In this way, our study suggested that adolescents who possessed lower SSS expectations in the future might not be able to withstand the stress elicited by higher parental expectations, thus impairing their persistence. In addition, the discrepancy between higher perceived parental expectations and lower adolescent future SSS expectations might reflect poor parent–child relationships and interactions [11], which could weaken adolescent persistence. Specifically, parental expectations for their children are not based on their children’s expectations [45]. Disparities in expectations between parents and their children are likely to lead to tense parent–child relationships [27,49]. Such adolescents may feel misunderstood by their parents, resulting in poor parent–child interactions, which cannot facilitate adolescents’ behavioral outcomes, such as persistence. As such, for adolescents with lower expected future SSS, high parental expectations might become a burden (e.g., higher stress and poor parent–child relationships) and thus not benefit to their persistence.

Taken together, the extant research has explored adolescents’ perceived parental expectations and their own expectations separately, as well as their contributions to adolescents’ motivational and behavioral outcomes [2,3,4]. The current study has extended these previous studies by taking a fuller perspective, which considers both adolescents’ perceived parental expectations and their own expectations simultaneously. Although previous studies revealed the contributions of parental expectations to adolescents’ several outcomes [27,28,29], this study did not find such positive effects with respect to rural adolescents’ persistence. Thus, the findings of this study go beyond the general context to focus on the rural context, which is more likely to be socioeconomically disadvantaged, and suggest that significant others’ (i.e., parents’) expectations have little positive effect on persistence. In this way, this study has highlighted the significance of adolescents’ own expectations of future SSS in predicting persistence in the rural context.

## 5. Limitations, Future Research and Educational Implications

Several limitations of this study should be addressed, including but not limited to the cross-sectional nature of this study, the measurement of expected future SSS and the sources of data reports. First, although this study targeted rural adolescents from a once poverty-stricken area in China, more objective information regarding family economic conditions should be provided in future research. In this way, a fuller picture of how much economic risk these participants were exposed to could be captured. Second, the cross-sectional nature of this study limits our understanding of the long-term effects among variables. Future longitudinal research can be employed to examine how these study variables relate over time and help reveal the interactions between the two sources of expectations in the long term. Third, parental expectations could be reported by parents rather than only by adolescents, and examining the discrepancy in parent–child expectations could reveal family processes in the rural context. Fourth, although the use of a single item to measure the expected future SSS has advantages in reducing the burden on adolescents and has been used in previous studies, no research has specifically tested its reliability and validity; thus, future empirical research is needed to explicitly verify its reliability. Finally, given the small effect size (*f*^2^ = 0.02) and relatively small sample size of the present study, whether the findings can be broadly generalized to a group of rural adolescents needs further exploration, possibly through large-database research or rigorous experimental research.

Further research on the following aspects can be conducted in the future to expand the present study. First, this study was conducted in a rural context. Given the rural–urban differences in cultural attitudes, future research can test whether there are differences in the associations of the study variables in the urban context. Second, this study indicates that perceived parental expectations may not be conducive to persistence in adolescence, a developmental stage characterized by an emphasis on autonomy [46]. Whether these findings are different during childhood, a developmental period that is more aligned with parental authority and requires less autonomy, should be further explored in younger children. Third, this study indicates that perceived parental expectation is negatively related to rural adolescents’ persistence when they have lower levels of expected future SSS. As mentioned earlier, poor parent–child interactions and perceived high stress may be mediating mechanisms, but this needs further exploration. Fourth, other factors that are conducive to persistence should be extensively explored to identify effective ways to cultivate rural adolescents’ persistence. This can include but is not limited to psychological factors (e.g., mindset, supportive parenting) [31,75] and can also be extended to the societal level, such as income inequality [76]. Finally, both expectations and values are important factors associated with persistence [1], and the current study echoes expectancy–value theory but focuses on only one aspect (i.e., expectations) of the theory in the rural context. Future research can be better grounded in different economic contexts (e.g., rural and urban contexts, socioeconomically advantaged and disadvantaged contexts) within the expectancy–value framework to reveal the complex interactions between expectations and values on behavioral outcomes.

These findings have significant implications for rural families. Shaped by Chinese culture [11,25], there is no doubt that parents hold high expectations for their children. However, social culture should not only emphasize the value of parents’ high expectations but also need to recognize the limitations of high parental expectations [77]. As this study indicated, when rural adolescents perceive that their parents have high expectations but that they have lower expectations for future SSS, they do not exhibit sustained effort and perseverance. As suggested previously [26], it is of practical value to support adolescents in how to be more perseverant rather than just expressing high expectations for adolescents. In addition, our findings also support the implications of education policy for poverty alleviation in China. For decades, the Chinese government has placed high value on and implemented various forms of psychological assistance for the promotion of “ambition” among rural youth, aiming to cultivate rural youth’s own motivations to overcome the poverty cycle. As supported by our study, higher expectations for future SSS are consistent with the notion of “ambition”, helping rural adolescents demonstrate greater perseverance. Thus, a key psychological factor in promoting the persistence of rural youth is to increase their expectations on SSS in the future. Furthermore, combined with the findings of previous research focused on younger children [78], the adult model’s persistent behaviors are crucial in cultivating persistence. As such, it may also be important for adult role models (e.g., parents) to demonstrate perseverance in their daily lives in regard to how to cultivate the persistence of rural adolescents. Compared with the intangible expectations that parents hold for their children, their actions are more practical. After seeing their parents’ actions, adolescents also rationally allocate more effort and perseverance.

## 6. Conclusions

Given the nonsignificant main effect of perceived parental expectation on persistence, this study suggests that rural adolescents’ perceived parental expectation may not be a relevant factor that contributes to persistence. In addition, perceived parental expectation is significantly and negatively related to persistence when rural adolescents’ future SSS is lower but is not significantly correlated with persistence when rural adolescents’ future SSS is higher. This implies the potentially adverse effects of high perceived parental expectations on rural adolescents’ persistence and that expected future SSS can alleviate this adverse relationship. Rural adolescents’ expectations for future SSS are helpful in facilitating their persistence. Future research can develop specific interventions targeting the expected future SSS to improve rural adolescents’ persistence in the face of difficulties.

## Figures and Tables

**Table 1:** Descriptive statistics for the study variables.

Variable	Score	Range	*M*	*SD*	Shapiro–Wilk	
					Value	*p*
Perceived parental expectation	continuous	1~6	4.52	1.01	0.972	<0.001
Persistence	continuous	1~5	2.74	0.97	0.971	<0.001
Expected future SSS	continuous	1~10	7.34	1.92	0.933	<0.001
Gender	dummy	—	0.45	0.50	—	—
Age	continuous	12~15	13.32	0.72	—	—

Note. Gender is a dummy variable (boy = 0, girl = 1) and thus is regarded as a continuous variable in the data analyses; SSS = subjective social status; *M* = mean, *SD* = standard deviation.

**Table 2:** Correlations for the main variables.

Variable	1	2	3	4
1. perceived parental expectation	—			
2. persistence	0.01	—		
3. expected future subjective social status	0.05	0.25 ***	—	
4. gender	−0.10 **	−0.21 ***	−0.05	—
5. age	−0.03	−0.04	0.04	−0.09 *

Note. *** *p* < 0.001, ** *p* < 0.01, and * *p* < 0.05; gender is a dummy variable (boy = 0, girl = 1).

**Table 3:** The moderation model for rural adolescents’ persistence.

Predictors	*β*	*SE*	*95% CI*
perceived parental expectation	−0.02	0.04	[−0.13, 0.08]
expected future SSS	0.24 ***	0.04	[0.14, 0.35]
perceived parental expectation × expected SSS	0.10 *	0.04	[0.02, 0.18]
gender	−0.19 ***	0.04	[−0.26, −0.11]
age	−0.08 *	0.03	[−0.14, −0.01]

Note. *** *p* < 0.001, and * *p* < 0.05. Gender is a dummy variable (boy = 0; girl = 1). All other continuous variables were transferred into z scores.

## Data Availability

The data that support the findings of this study are available on request from the authors.
